# Fertility intention and its affecting factors in China: A national cross-sectional survey

**DOI:** 10.1016/j.heliyon.2023.e13445

**Published:** 2023-02-03

**Authors:** Ze Xiang, Xinyue Zhang, Yiqi Li, Jiarui Li, Yinlin Wang, Yujia Wang, Wai-Kit Ming, Xinying Sun, Bin Jiang, Guanghua Zhai, Yibo Wu, Jian Wu

**Affiliations:** a Zhejiang University School of Medicine, Hangzhou, Zhejiang, 310009, China; b The Affiliated Stomatology Hospital, Hangzhou, Zhejiang, 310000, China; c Zhejiang University School of Stomatology & Key Laboratory of Oral Biomedical Research of Zhejiang Province, Hangzhou, Zhejiang, 310000, China; d College of Humanities and Social Sciences, Harbin Medical University, 157 Health Care Road, Nangang District, Harbin City, Heilongjiang Province, China; e Department of Infectious Diseases and Public Health, Jockey Club College of Veterinary Medicine and Life Sciences, City University of Hong Kong, Hong Kong; f School of Public Health, Peking University, 38 Xueyuan Road, Haidian District, Beijing, China; g Department of Laboratory Medicine, The Central Blood Station of Yancheng City, Yancheng 224000, China; h Department of Laboratory Medicine, The Affiliated Suzhou Hospital of Nanjing Medical University, Suzhou Municipal Hospital, Gusu School, Nanjing Medical University, 242 Guangji Road, Suzhou 215008, Jiangsu, China

**Keywords:** China, Fertility intention, Second-child, Affecting factors, Fertility policies, Cross-sectional survey

## Abstract

**Introduction:**

Low fertility rate has become an inevitable problem globally. Although current policies have a certain effect on promoting fertility and raising the birth rate, the overall effect is not obvious to meet the need. Therefore, the exploration of fertility intention and its affecting factors is extremely significant.

**Methods:**

This study collected demographic data and the intention of respondents to have a second children, which focused on the factors that could affect fertility issues. 11,031 respondents were divided into non-fertile group (n = 5062) and fertile group (n = 5969) according to whether they had children or not, and the fertility group (n = 5969) were divided into group with 1–2 children (n = 5293) and group with ≥3 children (n = 676) according to the number of children. Non-fertility respondents aged 26–40 (n = 1369) were divided to explore the factors affecting the second-children intention. Binary logistic regression analysis was used to determine the affecting factors.

**Results:**

It was revealed that gender [Male: OR: 0.60, 95% CI: 0.54–0.68], age [26–40: OR: 16.0, 95% CI: 13.4–19.1; 41–60: OR: 233.8, 95% CI: 186.7–292.6; >60: OR: 105.6, 95% CI: 77.1–144.6], political status [Partisans: OR: 0.48, 95% CI: 0.42–0.54], highest educational level [Middle school: OR: 0.21, 95% CI: 0.17–0.26; College degree or above: OR: 0.09, 95% CI: 0.08–0.11], whether having chronic disease [Yes: OR: 1.95, 95% CI: 1.60–2.38] and depression [Mild depression: OR: 0.63, 95% CI: 0.56–0.72; Moderate depression: OR: 0.43, 95% CI: 0.36–0.53; Moderate to severe depression: OR: 0.45, 95% CI: 0.35–0.57; Severe depression: OR: 0.50, 95% CI: 0.33–0.74] were important factors affecting fertility intention. We found that age [26–40: OR: 0.11, 95% CI: 0.08–0.15; 41–60: OR: 0.15, 95% CI: 0.12–0.18; >60: 0.81, 95% CI: 0.66–0.99], region [Central China: OR: 1.49, 95% CI: 1.20–1.86; Western China: OR: 1.75, 95% CI: 1.41–2.18], resident place [Urban: OR: 0.59, 95% CI: 0.49–0.72], per capita monthly household income [6001–12000: OR: 0.63, 95% CI:0.46–0.83; ≥12,000: OR: 1.83, 95% CI: 1.20–2.80], political status [Non-partisans: OR: 0.24, 95% CI: 0.09–0.69], highest educational level [Middle school: OR: 0.36, 95%CI: 0.27–0.46; College degree or above: OR: 0.22, 95% CI: 0.17–0.30] and anxiety [Moderate anxiety: OR: 1.39, 95% CI: 1.04–1.88; Severe anxiety: OR: 2.19, 95% CI: 1.26–3.80] were the main affecting factors for choosing the number of children. Furthermore, the second-children intention investigation in respondents aged 26–40 showed that gender [Male: OR: 2.06, 95% CI: 1.67–2.53], resident place [Urban: OR: 0.59, 95% CI: 0.49–0.72], per capita monthly household income [≥12,000: OR: 1.86, 95% CI: 1.23–2.82] and pressure [Severe pressure: OR: 0.54, 95% CI: 0.34–0.85] were the important factors.

**Conclusion:**

Region, educational level, psychological factors, income, political status and medical insurance were the important factors affecting the intention of fertility and the number of children. The government should take these factors into account when optimizing the existing policy.

## 1 Introduction

Fertility issues have always been a global concern, and it was estimated that between 48 million couples and 186 million individuals have infertility [1]. With the development of economy and society, the fertility rate shows a trend of negative correlation [2,3]. In developed countries, the potential adverse consequences of chronically below-replacement-level fertility rate have been widely discussed [4]. Vollset et al. predicted that population decline will exceed 50% from 2017 to 2100 [5]. Low fertility rate has become an inevitable problem globally [6].

In 1979, China introduced the one-child policy, which successfully controlled the rapid population growth and improved the health and welfare of women and children [7]. In recent years, the social structure in China has changed, and the degree of aging has become increasingly serious [8]. Faced with the shrinking workforce and rapidly aging population, Chinese government ended the one-child policy in 2015, and liberalized the two-child policy to promote the balanced development of the population [9]. However, the fertility rate in China has been declining every year since 2016. In 2020, it was reported that the number of births dropped by 580,000 and the birth rate was 10.48 per 1,000, which indicated that existing policies could not meet the need to increase the fertility rate [10]. Since 2021, Chinese government and many regions successively introduced policies to stimulate the birth of second and third children [11].

Tatum proposed that the quality of medical services and the cost of treatment are important factors affecting fertility in China [10]. Of note, social support was considered to have no substantial effect on the increase of fertility in Europe [12]. The effects of chronic diseases such as arthritis on male and female fertility were explored [13,14]. Even climate may have a certain impact on fertility [15,16]. Furthermore, Ezeh et al. suggested that the links between policy, social and economic conditions and fertility remain limited [17]. Therefore, the role of factors influencing fertility is unclear.

Nowadays, China is the most populous country worldwide. The exploration of the factors affecting fertility in China would have guiding significance for China and the world. In this cross-sectional study, we focused on the affecting factors of these issues based on whether to have children, the choice of number of children, and the second-child intention in non-fertility respondents of the appropriate age, aiming to provide new strategies for optimizing fertility policies around the world.

## 2 Methods

### 2.1 Design of study

We carried out this survey from July 2021 to September 2021. This survey involved a total of 120 cities. The capital cities of 22 provinces, the capital cities of 5 autonomous regions and 4 municipalities were directly included, and 2–6 other prefecture-level cities in each province and autonomous region were included by random number table method. According to the data results of the “Seventh National Census 2021” [18], samples were obtained based on gender, age and resident place in line with demographic data by quota samples of the citizens in 120 cities (quota properties: gender, age and resident place). Specific quota sampling criteria (every 100 samples) are as follows: (1) Gender: The ratio of male to female is close to 1:1; (2) Age: ≤18: 8 ± 5 (samples); 19–24: 12 ± 5; 25–30: 12 ± 5; 31–40: 16 ± 5; 41–50: 18 ± 5; 51–60: 18 ± 5; 51–60: 18 ± 5; 61–70: 10 ± 5; ≥71: 18 ± 5; (3) Resident place: The ratio of urban to rural areas is close to 3:2. The investigators and their teams were openly enrolled to obtain the samples, and at least 1 investigator (or team) was assigned in each city. The investigators and their teams collected questionnaires through a professional online questionnaire survey platform called wenjuanxing (https://www.wjx.cn/vm/QxCemrk.aspx#). The data in the survey questionnaires was imported into the wenjuanxing platform by the investigators and their teams, and a link was created. The investigators and their team shared the link with enrolled respondents, so the enrolled respondents could answer the questionnaires online. Every respondent had the right to refuse to answer the questionnaires during the survey after providing the informed consent. If the enrolled respondents could not answer the questionnaires online, the investigators and their teams would provide the one-to-one interview through literary and verbal communication.

The inclusion criteria in this study were as follows: (1) the age of respondents should exceed 12 years; (2) people who have the nationality of the People's Republic of China; (3) the annual travel time of people is ≤ 1 month; (4) people who volunteer to participate in the survey and sign the inform consent; (5) people who could understand each item in the survey questionnaires; (6) people who could answer the online questionnaires alone or through the interview with the investigators. The exclusion criteria were the following: (1) people who are inconvenient for movement, and have confusion and mental disorders; (2) people who are involved in similar survey at the same time; (3) people who refuse to cooperate.

This research proposal was approved by the Institutional Review Committee of Jinan university (approval number: JNUKY-2021-018). The participation in this survey in all respondents was voluntary.

### 2.2 Research instrument

This study focused on the factors that could affect fertility issues. Based on the collected information, this study mainly analyzed sociodemographic characteristics (gender, age, number of children, region, resident place, highest education level and so on), psychological indicators (pressure, depression and anxiety) and intention to have a second children.

We used a 3-item self-made scale to measure self-pressure in respondents. For each item, a number from 1 to 6 was inputted by respondents, which suggested stress levels on family and work. The total score of the summation scale was between 3 and 18. The higher the score, the more significant the stress perception. The pressure status of respondents was classified by total score. 3–6 was classified for mild stress, 7–15 for moderate pressure, and 16–18 for severe pressure. The Cronbach's alpha of self-pressure scale was 0.861.

We employed the Patient Health Questionnaire (PHQ-9) to evaluate the depressive disorders in respondents. Nine items were scored on their frequency from “not at all” to “nearly every day”. The final score was between 0 and 27. The higher the score, the greater the depressive symptoms [19]. PHQ-9 meets the criteria for the diagnosis of depression in DSM-V. The severity of depression symptoms was classified by total score. 0–4 was defined as no symptoms, 5–9 as mild symptoms, 10–14 as moderate symptoms, 15–19 moderate to severe symptoms, and 19–27 as severe symptoms. The Cronbach's alpha of PHQ-9 was 0.940.

The anxiety status was measured by Generalized Anxiety Disorde-7 (GAD-7). We included 7 items to evaluate the frequency of anxiety symptoms by scoring. Each item was scored 0–3 and the total score was between 0 and 21. The total score of 0–4 was classified for no symptoms, 5–9 for mild symptoms, 10–14 for moderate symptoms, and 15–21 for severe symptoms [20]. The Cronbach's alpha of GAD-7 was 0.955.

We used this question “What is your intention to have two children?” to explore the intention to have a second children. The answer options were “skip”, “not applicable/unwilling to answer”, “no intention at all”, “no intention”, “common”, “intention” and “strong intention”, respectively. Excluding “skip” and “not applicable/unwilling to answer” options, “no intention” and “no intention at all” were defined as no desire to have a second children, while “common”, “intention” and “strong intention” were defined as a desire to have a second children in this study.

### 2.3 Statistical analysis

SPSS 26.0 was used for statistical analysis. We firstly performed the Chi-square analysis of the studied variables. Then both significant and potential influencing factors were selected to be further incorporated into binary logistic regression analysis. Forward logistic regression method was used in binary logistic regression analysis. *P* < 0.05 was considered statistically significant.

## 3 Result

### 3.1 Baseline characteristics of 11,301 respondents

**Fig. 1:**
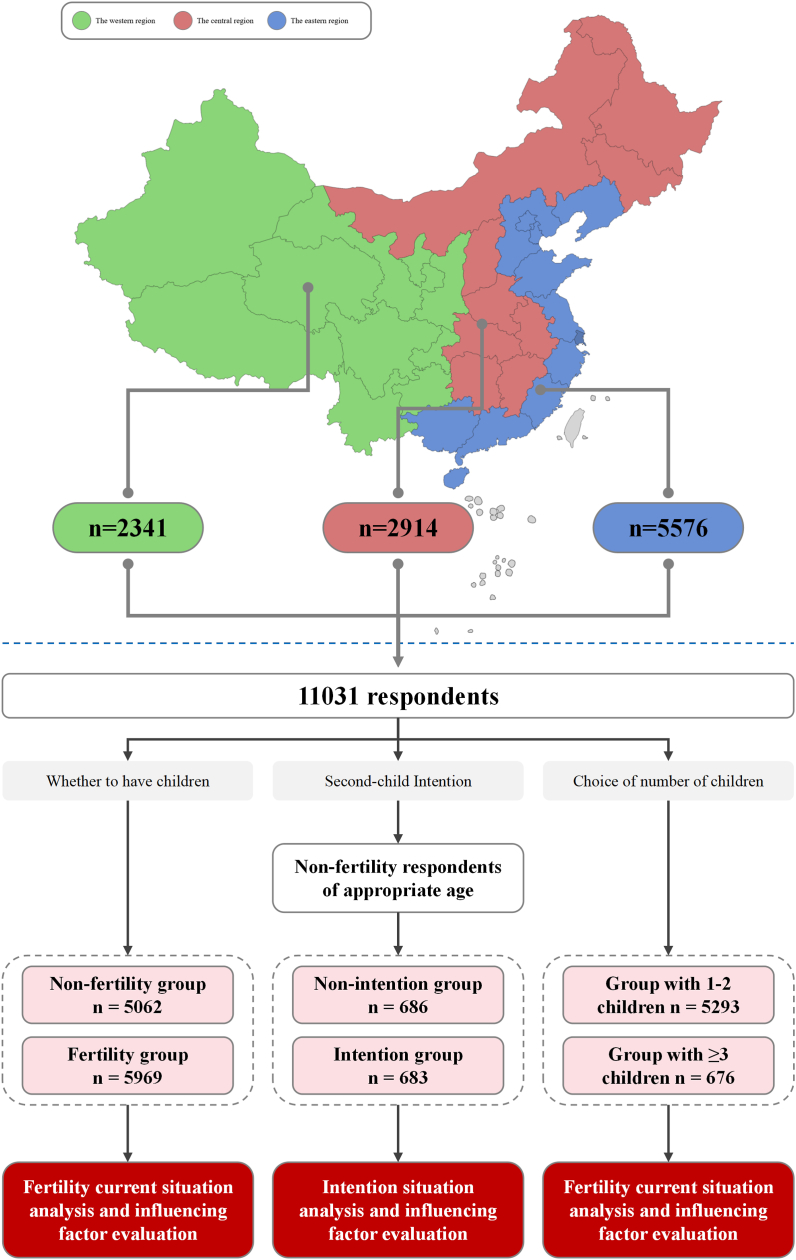
**The overall design of this study** This survey was carried out from July 2021 to September 2021. This survey involved 120 cities, including capital cities of 22 provinces, 5 autonomous regions and 4 municipalities, and 2–6 other prefecture-level cities selected by random number table method in each province and autonomous region. 50.9% of the respondents were from eastern China, while 25.8% and 23.3% were from central and western China. Firstly, according to whether they had children or not, 11,031 respondents were divided into non-fertile group (n = 5062) and fertile group (n = 5969) in order to study fertility intention and its affecting factors. Secondly, to explore the effect of the number of children on fertility, we divided the fertility group (n = 5969) into group with 1–2 children (n = 5293) and group with ≥3 children (n = 676) according to the number of children. Thirdly, to explore the factors affecting the second-child intention, we divided the remaining non-fertility respondents aged 26–40 into the non-intention group (n = 686) and the intention group (n = 683) according to whether they wanted to have a second child. Finally, we summarized the existing fertility promotion policies in China and gave suggestions that the government should take these factors into account when optimizing the fertility policies, especially region, educational level and psychological factors.

The overall design of this study is shown in Fig. 1. The baseline characteristics of 11,301 respondents recruited in this study was revealed in Table 1. Of the respondents, 54.4% were women. There were 3087, 3310, 3487 and 1147 respondents in the age ranges of ≤25, 26–40, 41–60 and > 60, respectively. 50.9% of the respondents were from eastern China, while 25.8% and 23.3% were from central and western China (Fig. 1). Most respondents (72.6%) were resident in urban areas. 47.4% of the respondents were partisans, a small number of respondents were non-partisans, and the rest were the masses. The highest educational level of 58.8% respondents surveyed was college degree or above.

**Table 1:** Baseline characteristics of all respondents.

Variables	N (%)
*Total*	11,031 (100.0)
*Gender*	
Female	5998 (54.4)
Male	5033 (45.6)
*Age group, year*	
≤25	3087 (28.0)
26-40	3310 (30.0)
41-60	3487 (31.6)
>60	1147 (10.4)
*Region*	
Eastern China	5611 (50.9)
Central China	2851 (25.8)
Western China	2569 (23.3)
*Resident place*	
Rural	3023 (27.4)
Urban	8008 (72.6)
*Per capita monthly household income*	
≤6000	7500 (68.0)
6001-12000	2769 (25.1)
>12,000	762 (6.9)
*Political status*	
The masses	5659 (51.3)
Non-partisans	146 (1.3)
Partisans	5226 (47.4)
*Highest educational level*	
Primary school or below	2566 (23.3)
Middle school	1978 (17.9)
College degree or above	6487 (58.8)
*Whether having chronic disease*	
No	2047 (18.6)
Yes	8984 (81.4)
*Depression*	
No depression	5031 (45.6)
Mild depression	3801 (34.5)
Moderate depression	1148 (10.4)
Moderate to severe depression	803 (7.3)
Severe depression	248 (2.2)
*Anxiety*	
No anxiety	6170 (55.9)
Mild anxiety	3364 (30.5)
Moderate anxiety	1198 (10.9)
Severe anxiety	299 (2.7)
*Pressure*	
Mild pressure	2719 (24.6)
Moderate pressure	7653 (69.4)
Severe pressure	659 (6.0)
*Number of children*	
0	5062 (45.9)
1-2	5293 (48.0)
≥3	676 (6.1)
*Intention to have two children*	
Skip	2212 (20.1)
Not applicable/unwilling to answer	2237 (20.3)
No intention at all	1598 (14.5)
No intention	1475 (13.4)
Common	2145 (19.4)
Intention	1050 (9.5)
Strong intention	314 (2.8)

45.9% of the respondents had no children, 48.0% had 1-2 children, and 6.1% had ≥3 children. The answers to whether you are willing have a second children were also shown in Table 1.

### 3.2 Fertility intention and its affecting factors

According to whether they had children or not, 11,031 respondents were divided into non-fertile group (n = 5062) and fertile group (n = 5969). Both significant and potential influencing factors were incorporated into binary logistic regression analysis. The results showed that gender, age, political status, highest educational level, whether having chronic disease and depression were significant in affecting fertility (all *P* < 0.05, Table 2).

**Table 2:** Logistic regression analysis between non-fertility group and fertility group.

Variables	Non-fertility group (n = 5062)	Fertility group (n = 5969)	Binary logistic regression OR (95% CI)	Binary logistic regression *P* value
*Gender*				
Female	2825 (55.8)	3173 (53.2)	Reference	
Male	2237 (44.2)	2796 (46.8)	0.60 (0.54–0.68)	<0.01
*Age group, year*				
≤25	3040 (60.0)	47 (0.8)	Reference	
26-40	1762 (34.8)	1548 (25.9)	16.0 (13.4–19.1)	<0.01
41-60	195 (3.9)	3292 (55.2)	233.8 (186.7–292.6)	<0.01
>60	65 (1.3)	1082 (18.1)	105.8 (77.1–144.6)	<0.01
*Region*				
Eastern China	2562 (50.6)	3049 (51.1)		
Central China	1298 (25.6)	1553 (26.0)		
Western China	1202 (23.8)	1367 (22.9)		
*Resident place*				
Rural	1335 (26.4)	1688 (28.3)		
Urban	3727 (73.6)	4281 (71.7)		
*Per capita monthly household income*				
≤6000	3368 (66.5)	4132 (69.2)		
6001-12000	1306 (25.8)	1463 (24.5)		
>12,000	388 (7.7)	374 (6.3)		
*Political status*				
The masses	1551 (30.6)	4108 (68.8)	Reference	
Non-partisans	38 (0.7)	108 (1.8)	0.73 (0.43–1.24)	0.25
Partisans	3473 (68.6)	1753 (29.4)	0.48 (0.42–0.54)	<0.01
*Highest educational level*				
Primary school or below	480 (9.5)	2086 (34.9)	Reference	
Middle school	718 (14.2)	1260 (21.1)	0.21 (0.17–0.26)	<0.01
College degree or above	3864 (76.3)	2623 (44.0)	0.09 (0.08–0.11)	<0.01
*Whether having chronic disease*				
No	4777 (94.4)	4207 (70.5)	Reference	
Yes	285 (5.6)	1762 (29.5)	1.95 (1.60–2.38)	<0.01
*Depression*				
No depression	2095 (41.4)	2936 (49.2)	Reference	
Mild depression	1741 (34.4)	2060 (34.5)	0.63 (0.56–0.72)	<0.01
Moderate depression	612 (12.1)	536 (9.0)	0.43 (0.36–0.53)	<0.01
Moderate to severe depression	464 (9.2)	339 (5.7)	0.45 (0.35–0.57)	<0.01
Severe depression	150 (2.9)	98 (1.6)	0.50 (0.33–0.74)	<0.01
*Anxiety*				
No anxiety	2667 (52.7)	3503 (58.7)		
Mild anxiety	1574 (31.1)	1790 (30.0)		
Moderate anxiety	642 (12.7)	556 (9.3)		
Severe anxiety	179 (3.5)	120 (2.0)		
*Pressure*				
Mild pressure	1217 (24.0)	1502 (25.2)		
Moderate pressure	3522 (69.6)	4131 (69.2)		
Severe pressure	323 (6.4)	336 (5.6)		

The proportion of males in the fertility group was lower than that in the non-fertility group [OR: 0.60, 95% CI: 0.54–0.68]. Compared with ≤25 years of age, the proportions of 26–40 years, 41–60 years and >60 years of age were much higher in the fertility group than those in the non-fertility group [26–40: OR: 16.0, 95% CI: 13.4–19.1; 41–60: OR: 233.8, 95% CI: 186.7–292.6; >60: OR: 105.6, 95% CI: 77.1–144.6]. Relative to the masses, the proportion of partisans was lower in the fertility group than that in the non-fertility group [OR: 0.48, 95% CI: 0.42–0.54], while there was no significant difference in the proportion of non-partisans between these two groups (*P* = 0.25). Compared with primary school or below, the proportions of middle school and college degree or above in the fertility group were lower than those in the non-fertility group [Middle school: OR: 0.21, 95% CI: 0.17–0.26; College degree or above: OR: 0.09, 95% CI: 0.08–0.11]. The proportion of chronic diseases was higher in the fertility group than in the non-fertility group [OR: 1.95, 95% CI: 1.60–2.38]. Compared with those with no depression, the proportions of mild, moderate, moderate to severe and severe depression in the fertility group were lower than those in the non-fertility group [Mild depression: OR: 0.63, 95% CI: 0.56–0.72; Moderate depression: OR: 0.43, 95% CI: 0.36–0.53; Moderate to severe depression: OR: 0.45, 95% CI: 0.35–0.57; Severe depression: OR: 0.50, 95% CI: 0.33–0.74].

### 3.3 Fertility intention with different numbers of children and its affecting factors

To explore the effect of the number of children on fertility, we further divided the fertility group (n = 5969) into group with 1–2 children (n = 5293) and group with ≥3 children (n = 676) according to the number of children.

Similarly, we firstly compared the non-fertility group (n = 5062) with group with 1–2 children (n = 5293). Both significant and potential influencing factors were incorporated into binary logistic regression analysis. The results revealed that gender, age, region, political status, highest educational level, whether having chronic disease and depression showed significant differences between these two groups (all *P* < 0.05, Table 3). Compared with eastern China, the proportions in central and western China were smaller in the group with 1–2 children than those in the non-fertility group [Central China: OR: 0.69, 95% CI: 0.60–0.80; Western China: OR: 0.59, 95% CI:0.51–0.68]. The comparison of remaining factors between group with 1–2 children and non-fertility group was consistent with the comparison between fertility group and non-fertility group.

**Table 3:** Logistic regression analysis between non-fertility group and group with 1–2 children.

Variables	Non-fertility group (n = 5062)	Group with 1–2 children (n = 5293)	Binary logistic regression OR (95% CI)	Binary logistic regression *P* value
*Gender*				
Female	2825 (55.8)	2809 (53.1)	Reference	
Male	2237 (44.2)	2484 (46.9)	0.62 (0.55–0.69)	<0.01
*Age group, year*				
≤25	3040 (60.0)	41 (0.8)	Reference	
26-40	1762 (34.8)	1482 (28.0)	17.2 (14.3–20.6)	<0.01
41-60	195 (3.9)	3075 (58.1)	254.2 (201.8–320.2)	<0.01
>60	65 (1.3)	695 (13.1)	91.3 (65.9–126.4)	<0.01
*Region*				
Eastern China	2562 (50.6)	2782 (52.6)	Reference	
Central China	1298 (25.6)	1361 (25.7)	0.69 (0.60–0.80)	<0.01
Western China	1202 (23.8)	1150 (21.7)	0.59 (0.51–0.68)	<0.01
*Resident place*				
Rural	1335 (26.4)	1333 (25.2)		
Urban	3727 (73.6)	3960 (74.8)		
*Per capita monthly household income*				
≤6000	3368 (66.5)	3564 (67.3)		
6001-12000	1306 (25.8)	1387 (26.2)		
>12,000	388 (7.7)	342 (6.5)		
*Political status*				
The masses	1551 (30.6)	3572 (67.5)	Reference	
Non-partisans	38 (0.7)	104 (2.0)	0.84 (0.50–1.42)	0.51
Partisans	3473 (68.6)	1617 (30.5)	0.50 (0.44–0.57)	<0.01
*Highest educational level*				
Primary school or below	480 (9.5)	1585 (29.9)	Reference	
Middle school	718 (14.2)	1174 (22.2)	0.24 (0.20–0.30)	<0.01
College degree or above	3864 (76.3)	2534 (47.9)	0.10 (0.08–0.12)	<0.01
*Whether having chronic disease*				
No	4777 (94.4)	3878 (73.3)	Reference	
Yes	285 (5.6)	1415 (26.7)	1.82 (1.49–2.23)	<0.01
*Depression*				
No depression	2095 (41.4)	2634 (49.8)	Reference	
Mild depression	1741 (34.4)	1812 (34.2)	0.66 (0.57–0.75)	<0.01
Moderate depression	612 (12.1)	467 (8.8)	0.44 (0.36–0.55)	<0.01
Moderate to severe depression	464 (9.2)	295 (5.6)	0.44 (0.34–0.55)	<0.01
Severe depression	150 (2.9)	85 (1.6)	0.45 (0.30–0.68)	<0.01
*Anxiety*				
No anxiety	2667 (52.7)	3138 (59.3)		
Mild anxiety	1574 (31.1)	1578 (29.8)		
Moderate anxiety	642 (12.7)	478 (9.0)		
Severe anxiety	179 (3.5)	99 (1.9)		
*Pressure*				
Mild pressure	1217 (24.0)	1331 (25.1)		
Moderate pressure	3522 (69.6)	3657 (69.1)		
Severe pressure	323 (6.4)	305 (5.8)		

Next, we compared group with 1–2 children (n = 5293) with group with ≥3 children (n = 676). As shown in Table 4, there were significant differences between the two groups in age, region, resident place, per capita monthly household income, political status, highest educational level and anxiety. Compared with ≤25 years of age, the proportions of 26–40 years, 41–60 years and >60 years of age were smaller in the group with ≥3 children than those in the group with 1–2 children [26–40: OR: 0.11, 95% CI: 0.08–0.15; 41–60: OR: 0.15, 95% CI: 0.12–0.18; >60: 0.81, 95% CI: 0.66–0.99]. Compared with eastern China, the proportions in the central and western China were larger in the group with ≥3 children than those in the group with 1–2 children [Central China: OR: 1.49, 95% CI: 1.20–1.86; Western China: OR: 1.75, 95% CI: 1.41–2.18]. The proportion of residents in urban areas in the group with ≥3 children was smaller than that in the group with 1–2 children [OR: 0.59, 95% CI: 0.49–0.72]. Compared with per capita monthly household income ≤6000, the proportion of per capita monthly income of 6001–12000 was smaller in the group with ≥3 children than that in the group with 1–2 children [OR: 0.63, 95% CI:0.46–0.83]. However, the proportion of per capita monthly household income ≥12,000 in the group with ≥3 children was larger than that in the group with 1–2 children [OR: 1.83, 95% CI: 1.20–2.80]. Compared with the masses, the proportion of non-partisans was lower in the group with ≥3 children than that in the group with 1–2 children [OR: 0.24, 95% CI: 0.09–0.69], while there existed no significant difference in the proportion of partisans between the two groups (*P* = 0.21). Compared with primary school or below, the proportions of middle school and college degree or above in the group with ≥3 children were lower than those in the group with 1–2 children [Middle school: OR: 0.36, 95%CI: 0.27–0.46; College degree or above: OR: 0.22, 95% CI: 0.17–0.30]. Compared with no anxiety, the proportions of moderate and severe anxiety in the group with ≥3 children were higher than those in the group with 1–2 children [Moderate anxiety: OR: 1.39, 95% CI: 1.04–1.88; Severe anxiety: OR: 2.19, 95% CI: 1.26–3.80], while no difference was found in mild anxiety between the two groups (*P* = 0.87).

**Table 4:** Logistic regression analysis between group with 1–2 children and group with ≥3 children.

Variables	Group with 1–2 children (n = 5293)	Group with ≥3 children (n = 676)	Binary logistic regression OR (95% CI)	Binary logistic regression *P* value
*Gender*				
Female	2809 (53.1)	364 (53.8)		
Male	2484 (46.9)	312 (46.2)		
*Age group, year*				
≤25	41 (0.8)	6 (0.9)	Reference	
26-40	1482 (28.0)	66 (9.8)	0.11 (0.08–0.15)	<0.01
41-60	3075 (58.1)	217 (32.1)	0.15 (0.12–0.18)	<0.01
>60	695 (13.1)	387 (57.2)	0.81 (0.66–0.99)	0.043
*Region*				
Eastern China	2782 (52.6)	267 (39.5)	Reference	
Central China	1361 (25.7)	192 (28.4)	1.49 (1.20–1.86)	<0.01
Western China	1150 (21.7)	217 (32.1)	1.75 (1.41–2.18)	<0.01
*Resident place*				
Rural	1333 (25.2)	355 (52.5)	Reference	
Urban	3960 (74.8)	321 (47.5)	0.59 (0.49–0.72)	<0.01
*Per capita monthly household income*				
≤6000	3564 (67.3)	568 (84.0)	Reference	
6001-12000	1387 (26.2)	76 (11.2)	0.63 (0.48–0.83)	<0.01
>12,000	342 (6.5)	32 (4.8)	1.83 (1.20–2.80)	<0.01
*Political status*				
The masses	3572 (67.5)	536 (79.3)	Reference	
Non-partisans	104 (2.0)	4 (0.6)	0.24 (0.09–0.69)	<0.01
Partisans	1617 (30.5)	136 (20.1)	1.17 (0.92–1.49)	0.21
*Highest educational level*				
Primary school or below	1585 (29.9)	501 (74.1)	Reference	
Middle school	1174 (22.2)	86 (12.7)	0.36 (0.27–0.46)	<0.01
College degree or above	2534 (47.9)	89 (13.2)	0.22 (0.17–0.30)	<0.01
*Whether having chronic disease*				
No	3878 (73.3)	329 (48.7)		
Yes	1415 (26.7)	347 (51.3)		
*Depression*				
No depression	2634 (49.8)	302 (44.7)		
Mild depression	1812 (34.2)	248 (36.7)		
Moderate depression	467 (8.8)	69 (10.2)		
Moderate to severe depression	295 (5.6)	44 (6.5)		
Severe depression	85 (1.6)	13 (1.9)		
*Anxiety*				
No anxiety	3138 (59.3)	365 (54.0)	Reference	
Mild anxiety	1578 (29.8)	212 (31.4)	1.02 (0.83–1.24)	0.87
Moderate anxiety	478 (9.0)	78 (11.5)	1.39 (1.04–1.88)	0.028
Severe anxiety	99 (1.9)	21 (3.1)	2.19 (1.26–3.80)	<0.01
*Pressure*				
Mild pressure	1331 (25.1)	171 (25.3)		
Moderate pressure	3657 (69.1)	474 (70.1)		
Severe pressure	305 (5.8)	31 (4.6)		

### 3.4 Second-child intention and its affecting factors

To explore the factors affecting the second-child intention, we selected non-fertility respondents aged 26–40 for further study (n = 1762). When asked whether they would like to have a second child, a total of 393 eligible respondents chose “skip” and “not applicable/unwilling to answer”. After weeding out the respondents who did not express a clear will, we divided the remaining non-fertility respondents aged 26–40 into the non-intention group (n = 686) and the intention group (n = 683) according to whether they wanted to have a second child (Table 5).

**Table 5:** Logistic regression analysis between non-intention group and intention group.

Variables	Non-intention group (n = 686)	Intention group (n = 683)	Binary logistic regression OR (95% CI)	Binary logistic regression *P* value
*Gender*				
Female	433 (63.1)	317 (46.4)	Reference	
Male	253 (36.9)	366 (53.6)	2.06 (1.67–2.53)	<0.01
*Region*				
Eastern China	348 (50.7)	344 (51.5)		
Central China	166 (24.2)	178 (27.2)		
Western China	172 (25.1)	161 (21.3)		
*Resident place*				
Rural	111 (16.2)	157 (23.0)	Reference	
Urban	575 (83.8)	526 (77.0)	0.59 (0.49–0.72)	<0.01
*Per capita monthly household income*				
≤6000	421 (61.4)	408 (59.7)	Reference	
6001-12000	223 (32.5)	202 (29.6)	0.99 (0.78–1.27)	0.94
>12,000	42 (6.1)	73 (10.7)	1.86 (1.23–2.82)	<0.01
*Political status*				
The masses	301 (43.9)	271 (39.7)		
Non-partisans	9 (1.3)	8 (1.2)		
Partisans	376 (54.8)	404 (59.2)		
*Highest educational level*				
Primary school or below	24 (3.5)	28 (4.1)		
Middle school	56 (8.2)	52 (7.6)		
College degree or above	606 (88.3)	603 (88.3)		
*Whether having chronic disease*				
No	636 (92.7)	639 (93.6)		
Yes	50 (7.3)	44 (6.4)		
*Depression*				
No depression	270 (39.4)	285 (41.7)		
Mild depression	241 (35.1)	228 (33.4)		
Moderate depression	101 (14.7)	71 (10.4)		
Moderate to severe depression	57 (8.3)	76 (11.1)		
Severe depression	17 (2.5)	23 (3.4)		
*Anxiety*				
No anxiety	352 (51.3)	342 (50.1)		
Mild anxiety	233 (34.0)	210 (30.7)		
Moderate anxiety	78 (11.4)	102 (14.9)		
Severe anxiety	23 (3.3)	29 (4.3)		
*Pressure*				
Mild pressure	123 (17.9)	174 (25.5)	Reference	
Moderate pressure	507 (73.9)	471 (69.0)	0.81 (0.66–1.00)	0.06
Severe pressure	56 (8.2)	38 (5.5)	0.54 (0.34–0.85)	<0.01

There were significant differences in gender, resident place, per capita monthly household income and pressure between the two groups (all *P* < 0.05). The proportion of males in the intention group was higher than that in the non-intention group [OR: 2.06, 95% CI: 1.67–2.53]. The proportion of residents in the urban areas in the intention group was smaller than that in the non-intention group [OR: 0.59, 95% CI: 0.49–0.72]. Compared to per capita monthly household income ≤6000, the proportion of per capita monthly household income ≥12,000 was larger in the intention group than that in the non-intention group [OR: 1.86, 95% CI: 1.23–2.82], while there existed no difference between the two groups in the per capita monthly household income of 6001–12000 (*P* = 0.94). Compared with mild pressure, the proportion of severe pressure in the intention group was lower than that in the non-intention group [OR: 0.54, 95% CI: 0.34–0.85], while no significant difference was observed in the proportion of moderate pressure between the two groups (*P* = 0.06).

## 4 Discussion

Fertility has always been the focus of discussion in China and even around the world. As the problem of aging in society becomes more and more serious, increasingly countries with low-birth rate have been working to promote fertility in an attempt to change the social structure of aging. Despite the efforts made by governments to enact policies to promote fertility, the increase in the birth rate is still not obvious. Therefore, it is extremely important to explore the factors that may affect the fertility. As the world's most populous country, China's exploration of the factors affecting the fertility problems would have guiding significance for all the world. In general, this cross-sectional study explored whether to have children, the choice of number of children and the intention to have a second children, which may provide new insights into how China and other countries could develop better fertility policies.

It was showed that gender, age, political status, highest educational level, whether having chronic disease and depression are common affecting factors for fertility and the number of children. Gender differences were consistent with the results of exploring the reproductive behavior of floating population in China [21]. The legal age for marriage in mainland China is 22 for men and 20 for women, and after the age of 25, married people gradually begin to have children. Therefore, in the 26–40, 41–60 and > 60 years old groups, there are more people who have already had children. Besides, people aged 26–40, 41–60 and > 60 had a higher proportion of 1–2 children, which may be related to the one-child policy. Differences in political status may be explained by the fact that the Chinese government's previous one-child policy has been actively called on by all partisans. High educational level differences in respondents also proved that educational level does affect fertility intention [22]. The relatively high incidence of having chronic diseases in the fertility group illustrated the inseparability of medical and reproductive issues. The Chinese government is committed to the construction of medical insurance, which is a wise choice worth continuing. Moreover, the degree of depression was also related to fertility. We considered that depression may be an influencing factor that declines the desire to have children, which may be ignored by current policies. It was demonstrated that mood disorders negatively affect fertility [23,24]. Therefore, the government should pay attention to the depression of the non-fertility population and provide them with appropriate guidance and treatment.

In addition, region, resident place, per capita monthly household income and anxiety may also be related to the number of children. Region differed significantly, which may be related to the customs and habits of the central and western China. People in the central and western China may be willing to have children than those in the eastern China [18]. The decline in fertility rate due to industrial factors should also be taken into account since the degree of industrialization varies widely in these regions [25]. Difference in residents place may be explained by that people living in higher socioeconomic areas are more likely to have fewer children [26]. The significant differences in region and resident place suggest that fertility policy formulation should be tailored to local conditions. Besides, the choice of the number of children is related to economic factors, people with higher income may be willing to have more children, which reveals that financial support could increase people's intention to have more children. Moreover, it is worth noting that accompany with the number of children increases, the level of anxiety may also increase. Hence, after the promulgation of the three-child policy, the government should pay attention to the anxiety level of those who have already given birth, especially those who have more children.

In 2011, Alcorn et al. raised questions about how many people would be willing to have a second child after the one-child policy was liberalized [8]. The one-child policy ended in 2015, and China liberalized the two-child policy. In 2016, the Women's Federation said 53.3% of one-child families had no intention of having a second child. In the survey, they also found that education, medical care, sanitation and living environment were important factors influencing the decision to have a second child. Nevertheless, to further promote fertility, the Chinese government enacted a three-child policy in 2021. Therefore, it is necessary to explore the intention of non-fertility respondents of the appropriate age to have a second child, which plays a key role in optimizing existing policies and promoting fertility. Hence, the factors affecting the second-child intention was further explored. The non-fertility respondents aged 26–40 were considered as the eligible population in our study. Significant differences were found in gender, resident status, per capita monthly household income and pressure between the non-intention and intention groups. Gender differences may indicate that the non-fertility men of appropriate age are more eager to have children than women. It was also proved that economic factors would affect the intention to have children. It is worth noting that the pressure level of those who have the intention of having a second child is relatively low. Nargund et al. concluded that pressure may be a pathogenic factor of male fertility [27]. Therefore, the degree of pressure is inseparable from the desire to have children. Here, we speculate that the lower the pressure level, the stronger the intention to have children.

**Fig. 2:**
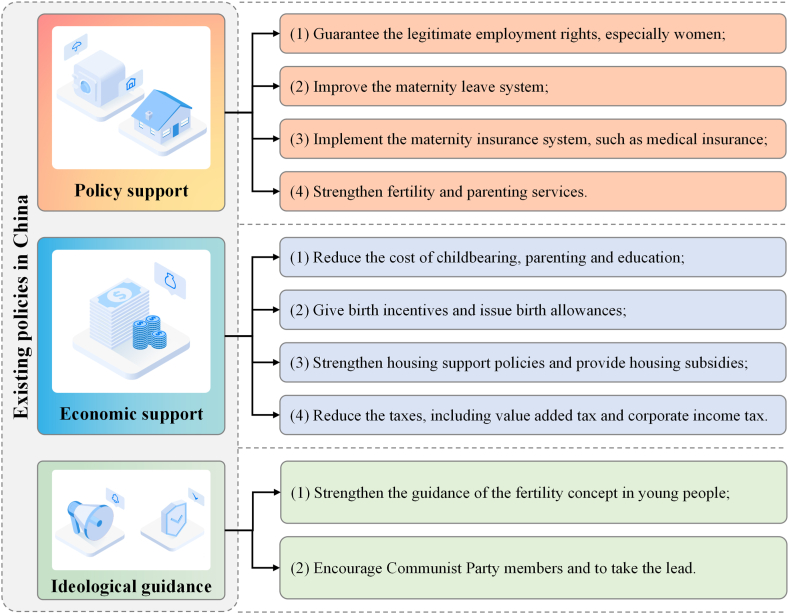
**The existing fertility promotion policies in China** The Chinese government has issued many fertility promotion policies recently, which mainly focus on policy support, economic support and ideological guidance.

Indeed, the Chinese government has issued many fertility promotion policies recently, which mainly focus on policy support, economic support and ideological guidance (Fig. 2). Whether having chronic disease is an important factor affecting fertility, and it is reflected in the fertility insurance in policy support. Economic factors such as income are important factors affecting the choice of number of children and fertility intention, and are reflected in economic subsidies and fertility incentives in economic support [28]. Moreover, political status is also an important factor affecting the choice of fertility and the number of children, which is reflected in the ideological guidance of encouraging Communist Party members and Communist Youth League members to take the lead to have children. Therefore, the existing policies have a certain effect on promoting fertility and raising the birth rate. However, the current policy effect is not obvious, because there are still other factors that need to be paid attention to, and the existing policy needs to be further optimized. On the existing basis, the government should take these factors into account when optimizing the fertility policies, especially region, educational level and psychological factors. Fertility policies should be formulated according to local conditions. Moreover, the government should also increase holidays to reduce the pressure of work, increase public psychological counseling clinics, and include maternal mental health counseling in the scope of medical insurance.

There exist some limitations in our study. Firstly, our study does not cover all cities in all China, which may lead to some incompleteness. Secondly, the distribution of the respondents was imbalanced, and the selecting bias of the study population may exist. Thirdly, our study does not use a family as a sampling unit, and more stuides are needed.

## 5 Conclusion

In conclusion, our study showed region, educational level, psychological factors, income, political status and medical insurance were the important factors affecting the choice of fertility and the number of children, which not only supported existing policies, but also provided recommendations for optimizing policies. Existing policies in China should continue to be implemented, including policy support, economic support and ideological guidance. Moreover, providing more psychological support is also recommended.

## Author contribution statement

Ze Xiang: Conceived and designed the experiments; Wrote the paper.

Xinyue Zhang: Performed the experiments; Wrote the paper.

Yiqi Li; Jiarui Li: Performed the experiments.

Yinlin Wang; Yujia Wang; Wai-Kit Ming: Analyzed and interpreted the data.

Xinying Sun; Bin Jiang; Guanghua Zhai: Contributed reagents, materials, analysis tools or data.

Yibo Wu; Jian Wu: Conceived and designed the experiments.

## Funding statement

This research did not receive any specific grant from funding agencies in the public, commercial, or not-for-profit sectors.

## Data availability statement

All data relevant to the study are included in the article.

## Declaration of interest's statement

The authors declare that they have no known competing financial interests or personal relationships that could have appeared to influence the work reported in this paper.

## Additional information

Supplementary content related to this article has been published online at [URL].
