# Supplementary material for: Fertility intention and its affecting factors in China: A national cross-sectional survey
Source: Heliyon. 2023 Feb 3;9(2):e13445. doi: 10.1016/j.heliyon.2023.e13445 (PMC9939585; doi:10.1016/j.heliyon.2023.e13445)
Supplement: Multimedia component 1 [file mmc1.docx]

**Full questionnaire involved in this study**

1. What is your gender?

○ Male ○ Female

2. What is your age range?

○ ≤25 ○ 26-40 ○ 41-60 ○ >60

3. Which city are you from?

4. What is your permanent residence?

○ Rural ○ Urban

5. What is your per capita monthly household income?

○ ≤6000 ○ 6001-12000 ○ >12000

6. What is your political status?

○ The masses ○ Non-partisans ○ Partisans

7. What is your highest educational level?

○ Primary school or below ○ Middle school ○ College degree or above

8. Do you have chronic diseases?

○ No ○ Yes

9. What is your number of children?

○ 0 ○ 1-2 ○ ≥3

10. How do you evaluate your ability to deal with stress? (1 means "I can get rid of stress" to 6 means "stress has been bothering me for a long time". Choose one of the numbers from 1 to 6)

11. In the past two weeks, how did you evaluate the stress in your life (including family and work)?

(1 means "no pressure" to 6 means "extreme pressure". Choose one of the numbers from 1 to 6)

12. In the past year, how did you evaluate the stress in your life (including family and work)?

(1 means "no pressure" to 6 means "extreme pressure". Choose one of the numbers from 1 to 6)

13. In the past two weeks, how often did you have the following symptoms in your life? [Matrix multiple choice questions]

|  | Not at all | For a few days | More than half | Almost every day |
| --- | --- | --- | --- | --- |
| Feeling nervous, anxious or anxious. | ○ | ○ | ○ | ○ |
| Unable to stop or control worry. | ○ | ○ | ○ | ○ |
| Worry too much about all kinds of things. | ○ | ○ | ○ | ○ |
| Hard to relax. | ○ | ○ | ○ | ○ |
| Unable to sit quietly because of anxiety. | ○ | ○ | ○ | ○ |
| Become easily annoyed or impatient. | ○ | ○ | ○ | ○ |
| Feel as if something terrible is going to happen and be afraid. | ○ | ○ | ○ | ○ |

14. In the past two weeks, have you often been troubled by the following questions? [Matrix multiple choice questions]

|  | Not at all | For a few days | More than half | Almost every day |
| --- | --- | --- | --- | --- |
| Do everything boring or don't want to do anything at all. | ○ | ○ | ○ | ○ |
| Feeling depressed, depressed or desperate. | ○ | ○ | ○ | ○ |
| It's hard to fall asleep and wake up in the middle of the night. Or, on the contrary, sleeping too much. | ○ | ○ | ○ | ○ |
| Feel tired or inactive. | ○ | ○ | ○ | ○ |
| Poor appetite or overeating | ○ | ○ | ○ | ○ |
| I don't like myself-I feel that I am doing badly, disappointed in myself or have negative family expectations. | ○ | ○ | ○ | ○ |
| It is difficult to concentrate on things, such as reading newspapers or watching TV. | ○ | ○ | ○ | ○ |
| Others report that you are slow to act or speak. Or, on the contrary, you are more active than usual-fidgeting and unable to stop. | ○ | ○ | ○ | ○ |
| I think I'd better die or hurt myself. | ○ | ○ | ○ | ○ |

15. What is your intention to have two children?

○ Skip

○ Not applicable/unwilling to answer

○ No intention at all

○ No intention

○ Common

○ Intention

○ Strong intention

**Table S1** **Chi-square test between non-fertility group and fertility group**

| Variables | Non-fertility group  (n = 5062) | Fertility group  (n = 5969) | Chi-square test  *P* value |  |
| --- | --- | --- | --- | --- |
| *Gender* | | | | |
| Female | 2825 (55.8) | 3173 (53.2) | < 0.01 |  |
| Male | 2237 (44.2) | 2796 (46.8) |  |  |
| *Age group, year* | | | | |
| ≤25 | 3040 (60.0) | 47 (0.8) | < 0.01 |  |
| 26-40 | 1762 (34.8) | 1548 (25.9) |  |  |
| 41-60 | 195 (3.9) | 3292 (55.2) |  |  |
| >60 | 65 (1.3) | 1082 (18.1) |  |  |
| *Region* | | | | |
| Eastern China | 2562 (50.6) | 3049 (51.1) | 0.58 |  |
| Central China | 1298 (25.6) | 1553 (26.0) |  |  |
| Western China | 1202 (23.8) | 1367 (22.9) |  |  |

**Table S1-continued**

| Variables | Non-fertility group  (n = 5062) | Fertility group  (n = 5969) | Chi-square test  *P* value |  |
| --- | --- | --- | --- | --- |
| *Resident place* | | | | |
| Rural | 1335 (26.4) | 1688 (28.3) | 0.03 |  |
| Urban | 3727 (73.6) | 4281 (71.7) |  |  |
| *Per capita monthly household income* | | | | |
| ≤6000 | 3368 (66.5) | 4132 (69.2) | < 0.01 |  |
| 6001-12000 | 1306 (25.8) | 1463 (24.5) |  |  |
| >12000 | 388 (7.7) | 374 (6.3) |  |  |
| *Political status* | | | | |
| The masses | 1551 (30.6) | 4108 (68.8) | < 0.01 |  |
| Non-partisans | 38 (0.7) | 108 (1.8) |  |  |
| Partisans | 3473 (68.6) | 1753 (29.4) |  |  |
| *Highest educational level* | | | |  |

**Table S1-continued**

| Variables | Non-fertility group  (n = 5062) | Fertility group  (n = 5969) | Chi-square test  *P* value |  |
| --- | --- | --- | --- | --- |
| Primary school or below | 480 (9.5) | 2086 (34.9) | < 0.01 |  |
| Middle school | 718 (14.2) | 1260 (21.1) |  |  |
| College degree or above | 3864 (76.3) | 2623 (44.0) |  |  |
| *Whether having chronic disease* | | | | |
| No | 4777 (94.4) | 4207 (70.5) | < 0.01 |  |
| Yes | 285 (5.6) | 1762 (29.5) |  |  |
| *Depression* | | | | |
| No depression | 2095 (41.4) | 2936 (49.2) | < 0.01 |  |
| Mild depression | 1741 (34.4) | 2060 (34.5) |  |  |
| Moderate depression | 612 (12.1) | 536 (9.0) |  |  |
| Moderate to severe depression | 464 (9.2) | 339 (5.7) |  |  |
| Severe depression | 150 (2.9) | 98 (1.6) |  |  |

**Table S1-continued**

| Variables | Non-fertility group  (n = 5062) | Fertility group  (n = 5969) | Chi-square test  *P* value |  |
| --- | --- | --- | --- | --- |
| *Anxiety* | | | | |
| No anxiety | 2667 (52.7) | 3503 (58.7) | < 0.01 |  |
| Mild anxiety | 1574 (31.1) | 1790 (30.0) |  |  |
| Moderate anxiety | 642 (12.7) | 556 (9.3) |  |  |
| Severe anxiety | 179 (3.5) | 120 (2.0) |  |  |
| *Pressure* | | | | |
| Mild pressure | 1217 (24.0) | 1502 (25.2) | 0.13 |  |
| Moderate pressure | 3522 (69.6) | 4131 (69.2) |  |  |
| Severe pressure | 323 (6.4) | 336 (5.6) |  |  |

**Table S2** **Chi-square test between non-fertility group and group with 1-2 children**

| Variables | Non-fertility group  (n = 5062) | Group with 1-2 children  (n = 5293) | Chi-square test  *P* value |  |
| --- | --- | --- | --- | --- |
| *Gender* | | | | |
| Female | 2825 (55.8) | 2809 (53.1) | < 0.01 |  |
| Male | 2237 (44.2) | 2484 (46.9) |  |  |
| *Age group, year* | | | | |
| ≤25 | 3040 (60.0) | 41 (0.8) | < 0.01 |  |
| 26-40 | 1762 (34.8) | 1482 (28.0) |  |  |
| 41-60 | 195 (3.9) | 3075 (58.1) |  |  |
| >60 | 65 (1.3) | 695 (13.1) |  |  |
| *Region* | | | | |
| Eastern China | 2562 (50.6) | 2782 (52.6) | 0.038 |  |
| Central China | 1298 (25.6) | 1361 (25.7) |  |  |
| Western China | 1202 (23.8) | 1150 (21.7) |  |  |

**Table S2-continued**

| Variables | Non-fertility group  (n = 5062) | Group with 1-2 children  (n = 5293) | Chi-square test  *P* value |  |
| --- | --- | --- | --- | --- |
| *Resident place* | | | | |
| Rural | 1335 (26.4) | 1333 (25.2) | 0.17 |  |
| Urban | 3727 (73.6) | 3960 (74.8) |  |  |
| *Per capita monthly household income* | | | | |
| ≤6000 | 3368 (66.5) | 3564 (67.3) | 0.057 |  |
| 6001-12000 | 1306 (25.8) | 1387 (26.2) |  |  |
| >12000 | 388 (7.7) | 342 (6.5) |  |  |
| *Political status* | | | | |
| The masses | 1551 (30.6) | 3572 (67.5) | < 0.01 |  |
| Non-partisans | 38 (0.7) | 104 (2.0) |  |  |
| Partisans | 3473 (68.6) | 1617 (30.5) |  |  |
| *Highest educational level* | | | |  |

**Table S2-continued**

| Variables | Non-fertility group  (n = 5062) | Group with 1-2 children  (n = 5293) | Chi-square test  *P* value |  |
| --- | --- | --- | --- | --- |
| Primary school or below | 480 (9.5) | 1585 (29.9) | < 0.01 |  |
| Middle school | 718 (14.2) | 1174 (22.2) |  |  |
| College degree or above | 3864 (76.3) | 2534 (47.9) |  |  |
| *Whether having chronic disease* | | | | |
| No | 4777 (94.4) | 3878 (73.3) | < 0.01 |  |
| Yes | 285 (5.6) | 1415 (26.7) |  |  |
| *Depression* | | | | |
| No depression | 2095 (41.4) | 2634 (49.8) | < 0.01 |  |
| Mild depression | 1741 (34.4) | 1812 (34.2) |  |  |
| Moderate depression | 612 (12.1) | 467 (8.8) |  |  |
| Moderate to severe depression | 464 (9.2) | 295 (5.6) |  |  |
| Severe depression | 150 (2.9) | 85 (1.6) |  |  |

**Table S2-continued**

| Variables | Non-fertility group  (n = 5062) | Group with 1-2 children  (n = 5293) | Chi-square test  *P* value |  |
| --- | --- | --- | --- | --- |
| *Anxiety* | | | | |
| No anxiety | 2667 (52.7) | 3138 (59.3) | < 0.01 |  |
| Mild anxiety | 1574 (31.1) | 1578 (29.8) |  |  |
| Moderate anxiety | 642 (12.7) | 478 (9.0) |  |  |
| Severe anxiety | 179 (3.5) | 99 (1.9) |  |  |
| *Pressure* | | | | |
| Mild pressure | 1217 (24.0) | 1331 (25.1) | 0.22 |  |
| Moderate pressure | 3522 (69.6) | 3657 (69.1) |  |  |
| Severe pressure | 323 (6.4) | 305 (5.8) |  |  |

**Table S3** **Chi-square test between group with 1-2 children and group with ≥3 children**

| Variables | Group with 1-2 children  (n = 5293) | Group with ≥3 children  (n = 676) | Chi-square test  *P* value |  |
| --- | --- | --- | --- | --- |
| *Gender* | | | | |
| Female | 2809 (53.1) | 364 (53.8) | 0.70 |  |
| Male | 2484 (46.9) | 312 (46.2) |  |  |
| *Age group, year* | | | | |
| ≤25 | 41 (0.8) | 6 (0.9) | < 0.01 |  |
| 26-40 | 1482 (28.0) | 66 (9.8) |  |  |
| 41-60 | 3075 (58.1) | 217 (32.1) |  |  |
| >60 | 695 (13.1) | 387 (57.2) |  |  |
| *Region* | | | | |
| Eastern China | 2782 (52.6) | 267 (39.5) | < 0.01 |  |
| Central China | 1361 (25.7) | 192 (28.4) |  |  |
| Western China | 1150 (21.7) | 217 (32.1) |  |  |

**Table S3-continued**

| Variables | Group with 1-2 children  (n = 5293) | | Group with ≥3 children  (n = 676) | Chi-square test  *P* value |  |
| --- | --- | --- | --- | --- | --- |
| *Resident place* | | | | | |
| Rural | | 1333 (25.2) | 355 (52.5) | < 0.01 |  |
| Urban | | 3960 (74.8) | 321 (47.5) |  |  |
| *Per capita monthly household income* | | | | | |
| ≤6000 | | 3564 (67.3) | 568 (84.0) | < 0.01 |  |
| 6001-12000 | | 1387 (26.2) | 76 (11.2) |  |  |
| >12000 | | 342 (6.5) | 32 (4.8) |  |  |
| *Political status* | | | | | |
| The masses | | 3572 (67.5) | 536 (79.3) | < 0.01 |  |
| Non-partisans | | 104 (2.0) | 4 (0.6) |  |  |
| Partisans | | 1617 (30.5) | 136 (20.1) |  |  |
| *Highest educational level* | | | | |  |

**Table S3-continued**

| Variables | Group with 1-2 children  (n = 5293) | | Group with ≥3 children  (n = 676) | Chi-square test  *P* value |  |
| --- | --- | --- | --- | --- | --- |
| Primary school or below | | 1585 (29.9) | 501 (74.1) | < 0.01 |  |
| Middle school | | 1174 (22.2) | 86 (12.7) |  |  |
| College degree or above | | 2534 (47.9) | 89 (13.2) |  |  |
| *Whether having chronic disease* | | | | | |
| No | | 3878 (73.3) | 329 (48.7) | < 0.01 |  |
| Yes | | 1415 (26.7) | 347 (51.3) |  |  |
| *Depression* | | | | | |
| No depression | | 2634 (49.8) | 302 (44.7) | 0.15 |  |
| Mild depression | | 1812 (34.2) | 248 (36.7) |  |  |
| Moderate depression | | 467 (8.8) | 69 (10.2) |  |  |
| Moderate to severe depression | | 295 (5.6) | 44 (6.5) |  |  |
| Severe depression | | 85 (1.6) | 13 (1.9) |  |  |

**Table S3-continued**

| Variables | Group with 1-2 children  (n = 5293) | | Group with ≥3 children  (n = 676) | Chi-square test  *P* value |  |
| --- | --- | --- | --- | --- | --- |
| *Anxiety* | | | | | |
| No anxiety | | 3138 (59.3) | 365 (54.0) | < 0.01 |  |
| Mild anxiety | | 1578 (29.8) | 212 (31.4) |  |  |
| Moderate anxiety | | 478 (9.0) | 78 (11.5) |  |  |
| Severe anxiety | | 99 (1.9) | 21 (3.1) |  |  |
| *Pressure* | | | | | |
| Mild pressure | | 1331 (25.1) | 171 (25.3) | 0.46 |  |
| Moderate pressure | | 3657 (69.1) | 474 (70.1) |  |  |
| Severe pressure | | 305 (5.8) | 31 (4.6) |  |  |

**Table S4** **Chi-square test between non-intention group and intention group**

| Variables | Non-intention group  (n = 686) | Intention group  (n = 683) | Chi-square test  *P* value |  |
| --- | --- | --- | --- | --- |
| *Gender* | | | | |
| Female | 433 (63.1) | 317 (46.4) | < 0.01 |  |
| Male | 253 (36.9) | 366 (53.6) |  |  |
| *Region* | | | | |
| Eastern China | 348 (50.7) | 344 (51.5) | 0.67 |  |
| Central China | 166 (24.2) | 178 (27.2) |  |  |
| Western China | 172 (25.1) | 161 (21.3) |  |  |
| *Resident place* | | | | |
| Rural | 111 (16.2) | 157 (23.0) | < 0.01 |  |
| Urban | 575 (83.8) | 526 (77.0) |  |  |
| *Per capita monthly household income* | | | |  |

**Table S4-continued**

| Variables | Non-intention group  (n = 686) | | Intention group  (n = 683) | Chi-square test  *P* value |  |
| --- | --- | --- | --- | --- | --- |
| ≤6000 | | 421 (61.4) | 408 (59.7) | < 0.01 |  |
| 6001-12000 | | 223 (32.5) | 202 (29.6) |  |  |
| >12000 | | 42 (6.1) | 73 (10.7) |  |  |
| *Political status* | | | | | |
| The masses | | 301 (43.9) | 271 (39.7) | 0.27 |  |
| Non-partisans | | 9 (1.3) | 8 (1.2) |  |  |
| Partisans | | 376 (54.8) | 404 (59.2) |  |  |
| *Highest educational level* | | | | | |
| Primary school or below | | 24 (3.5) | 28 (4.1) | 0.80 |  |
| Middle school | | 56 (8.2) | 52 (7.6) |  |  |
| College degree or above | | 606 (88.3) | 603 (88.3) |  |  |
| *Whether having chronic disease* | | | | |  |

**Table S4-continued**

| Variables | Non-intention group  (n = 686) | | Intention group  (n = 683) | Chi-square test  *P* value |  |
| --- | --- | --- | --- | --- | --- |
| No | | 636 (92.7) | 639 (93.6) | 0.54 |  |
| Yes | | 50 (7.3) | 44 (6.4) |  |  |
| *Depression* | | | | | |
| No depression | | 270 (39.4) | 285 (41.7) | 0.048 |  |
| Mild depression | | 241 (35.1) | 228 (33.4) |  |  |
| Moderate depression | | 101 (14.7) | 71 (10.4) |  |  |
| Moderate to severe depression | | 57 (8.3) | 76 (11.1) |  |  |
| Severe depression | | 17 (2.5) | 23 (3.4) |  |  |
| *Anxiety* | | | | | |
| No anxiety | | 352 (51.3) | 342 (50.1) | 0.16 |  |
| Mild anxiety | | 233 (34.0) | 210 (30.7) |  |  |
| Moderate anxiety | | 78 (11.4) | 102 (14.9) |  |  |
| Severe anxiety | | 23 (3.3) | 29 (4.3) |  |  |

**Table S4-continued**

| Variables | Non-intention group  (n = 686) | | Intention group  (n = 683) | Chi-square test  *P* value |  |
| --- | --- | --- | --- | --- | --- |
| *Pressure* | | | | | |
| Mild pressure | | 123 (17.9) | 174 (25.5) | < 0.01 |  |
| Moderate pressure | | 507 (73.9) | 471 (69.0) |  |  |
| Severe pressure | | 56 (8.2) | 38 (5.5) |  |  |
